# A collaboration to harmonize COVID-19 health messaging and fill communication gaps during initial U.S. refugee resettlement

**DOI:** 10.1080/28355245.2024.2311402

**Published:** 2024

**Authors:** Megan Keaveney, Cassie Le, Kate Steger, Neha J. Sood, Leticia Bligh, Curi Kim, SUSAn dicker, Alexander Klosovsky, Holly Herrera, Emily Jentes

**Affiliations:** a Centers for Disease Control and Prevention, Atlanta, Georgia, USA; b Department of State Bureau for Population, Refugees, and Migration, Washington, District of Columbia, USA; c International Rescue Committee, Washington, District of Columbia, USA; d Health Resources and Services Administration, Washington, District of Columbia, USA; e International Organization for Migration, Washington, District of Columbia, USA

**Keywords:** Refugee health, immigrant health, COVID-19, health communications

## Abstract

To communicate with U.S.-bound refugees during travel to the United States during the onset of the COVID-19 pandemic, five federal and international organizations collaborated in a strategic work group to synergize COVID-19 prevention health messaging and COVID-19 considerations before, during, and after travel, as well as promote shared resources. This work group sought to establish consistent COVID-19 messaging, disseminate messages to partners, and identify message gaps as the pandemic evolved. In early Fall 2020, CDC released new communication materials, including a fact sheet, a welcome booklet, and infographics translated into 19 languages, to address refugee health partners’ need for culturally and linguistically concordant educational materials for refugees. Rapidly changing health communications needs during the pandemic fostered opportunities for collaboration among federal and refugee health partners and highlighted a long-standing need among agencies to address health messaging across the continuum of care for refugees.

## Introduction

The COVID-19 pandemic was described as a “force multiplier,” increasing both the public health needs of refugees and immigrants while also making these needs more difficult to address (UNHCR The UN Refugee Agency, 2020). The pandemic also disproportionally affected refugee and immigrant communities, both abroad and domestically (Clarke et al., 2020). Ensuring the safety and well-being of refugee communities presents several challenges, including addressing issues of health equity (Shadmi et al., 2020). Each year, tens of thousands of refugees resettle to the United States; nearly 830,000 refugees resettled in the United States from 2008 to 2022 (Refugee Processing Center, 2022). Upon arrival, refugees are eligible for U.S. government–funded resettlement assistance, including healthcare benefits (Refugee Processing Center, 2022). Navigating the U.S. healthcare system for refugees poses several different challenges, including limited English proficiency, a social determinant that may serve as a barrier to accessing healthcare effectively. (Feinberg I. et al., 2020). In healthcare settings, refugee patients navigate both the instrumental and affective needs of ensuring that they both understand the health information presented to them but also feel like their needs and questions are fully heard and understood. (Noordman, J. et al., 2020).

Data on the impact of COVID-19 on the lives of resettled refugees remains limited. However, recently resettled refugees may be at a greater risk of contracting COVID-19 (Clarke et al., 2020). These populations face unique challenges to managing risk and prevention of COVID-19 due to a variety of factors including barriers to accessing COVID-19 testing and vaccination services; living in multigenerational housing where quarantine and isolation measures may be more difficult to maintain; being employed in settings with higher risk of exposure, such as service-sector jobs; experiencing language and healthcare access barriers; and having higher rates of co-morbidities (Centers for Disease Control and Prevention, 2021).

Although the U.S. refugee resettlement program was suspended from March to July 2020, a few refugees continued to travel due to concerns about their security. Resettlement staff responded rapidly to the situation amidst several critical challenges, including the sudden rollout of remote work protocols and newly created protocols for safe, in-person service delivery or remote service delivery whenever possible. Refugees also needed culturally and linguistically appropriate translations of COVID-19 prevention materials to communicate complex information and encourage adoption of public health prevention measures. Refugee health partners across the various federal agencies were responding to technical assistance requests for consistent and clear COVID-19 health messaging for refugees. This was the result of a growing realization and acknowledgement that several critical public health guidance were not tailored to the on-going needs of refugee communities, particularly at the beginning of the COVID-19 pandemic.

To address these ongoing challenges of COVID-19 prevention efforts within the refugee migration continuum of care, five organizations (Bureau of Population, Refugees and Migration [PRM]; Centers for Disease Control and Prevention [CDC]; Cultural Orientation Resource Exchange [CORE]; International Organization for Migration [IOM]; and the Administration for Children and Families’ Office of Refugee Resettlement [ORR]) formed a strategic work group to synergize COVID-19 health messaging and promote shared resources, establish a consistent COVID-19 messaging system, disseminate messages to partners, and identify gaps in health communications that could be addressed as the pandemic evolved and needs changed. Several key lessons were learned throughout the evolution of this work group regarding how to strengthen communication, collaboration, and coordination among refugee health partners at the international and federal level, which are highlighted throughout this paper. The aims of the work group, namely the development and distribution of COVID-19 health communications messaging, were achieved through enhanced coordination between federal partners; the widening distribution of communication networks; and the increased trust and accountability between federal and state partners to ensure health messaging and resources were distributed to refugee communities in their respective jurisdictions. These lessons serve as a blueprint for increased federal partner coordination of refugee health messaging for COVID-19 and future emergency responses.

## Methods

From March to December 2020, the work group met regularly to outline and discuss the communication and health recommendation gaps in the refugee continuum of care. Approximately 17 work group members represented across the 5 agencies, were active participants in the work group. In total, 11 meetings, each lasting about one hour’s duration, with all partners were held; smaller groups met more often to follow up on specific needs identified. In the smaller group discussions, efforts were made to ensure that at least one member of each agency participated. The work group was primarily facilitated by CDC staff, as the public health authority among the federal agency partners. While one individual would moderate the discussion, extensive notes were taken through a tracking spreadsheet that catalogued existing resources, gaps identified in health messaging, resource product updates, as well as forecasted distribution channels. The work group relied heavily on this spreadsheet for notetaking and tracking purposes. Although the work group did not conduct systematic data collection on communication gaps, informal data collection strategies were implemented through gathering critical feedback from partner networks to identify key communication gaps to provide rapid COVID-19 prevention messaging to refugees. During each meeting, participants from the agencies would share feedback from their networks, which included resettlement staff, public health practitioners, state refugee health coordinators, and refugee clients. Because the public health needs and guidance changed so rapidly with new information during the early stages of the pandemic, this feedback was critical to guiding the tasks and output of the work group. The work group also used experiences and communication templates from previous public health outbreak responses to assist in the development of resources (Joseph et al., 2019). Work group consensus on what kind of resources needed to be developed in addition to which agency would produce the resource was discussed at these meetings and tracked. After identifying messaging and resource gaps, the work group designated appropriate partners to design and develop these health communication resources. Dissemination strategy for the resources was determined through each work group partner, who respectively identified the existing platforms that would be best suited for widest dissemination. Each partner has a critical role in the migration continuum of care for refugees. By ensuring that the resources were distributed across the networks, refugees would be able to view the resources at multiple timepoints throughout their resettlement journey.

## Results

The work group identified four key communication gaps in translated COVID-19 prevention messaging across the migration continuum, which centered on: refugees with increased risk for severe illness from COVID-19; what COVID-19 prevention measures refugees needed to follow within the first two weeks after arrival in the United States; how refugees should contact their case management team about medical appointments and COVID-19 prevention after arrival; and how to integrate COVID-19 prevention education into domestic cultural orientation programs. The communication and resource gaps the group identified, and the resources the group developed are described in Table 1.

To address specific COVID-19 prevention measures for refugees at increased risk, CDC created a factsheet entitled “What You Can Do If You Are at Increased Risk of Severe Illness from COVID-19” with specific public health considerations tailored to refugee populations during movement from overseas to the United States, that could be distributed to refugees during their overseas medical visits. This factsheet defined who was at higher risk for severe illness from COVID-19 and specific tips for prevention before, during, and after travel to the United States. Although this messaging would be given to refugees at their required overseas medical exam, these topics could also be reiterated at the pre-departure medical checks within 48–72 hours before departure to the United States. The images and the wording used mirrored other CDC communication materials provided.

To further expand on CDC COVID-19 prevention measures for refugees, and with input from work group partners, CDC led the design and creation of a “Welcome Booklet” that could be shared with refugees before travel in addition to post-arrival by local refugee health partners and resettlement agency staff. This resource was developed based on domestic resettlement partner feedback on key communication needs and CDC recommendations. The goal of the Welcome Booklet was to provide consistent and reiterative COVID-19 prevention messaging on what refugees should do to protect themselves during the initial two weeks after arrival into the United States. The resource also included a space for resettlement agencies to provide contact information for the refugees’ case manager and instructions for obtaining medical assistance if needed. The images and the wording mirrored those of other communication materials provided. New content focused on COVID-19 vaccine knowledge, uptake, and, as well as COVID-19 testing on site and at-home, and other COVID-19 mitigation efforts were added to the resources to reflect updated changes to public health guidance. A CDC-funded project called the National Resource Center for Refugees, Immigrants, and Migrants (NRC-RIM) updated the Welcome Booklet in 2023 in the 19 original languages as well. (National Resource Center for Refugees, Immigrants, and Migrants, 2023).

**Figure 1.**
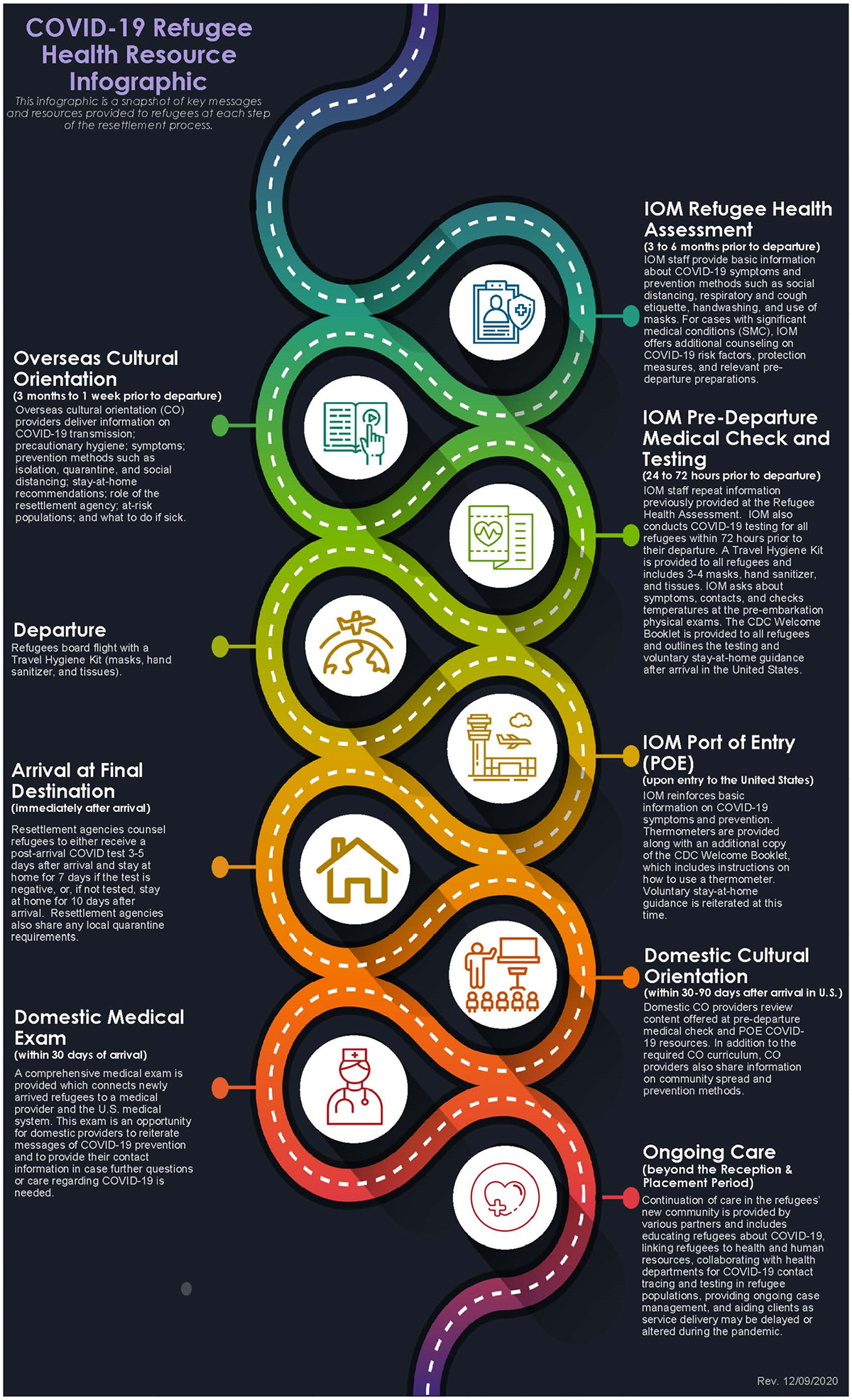
COVID-19 refugee health resource infographic developed by Bureau of populations, refugees, and Migration (Archived June 2022).

**Figure 2.**
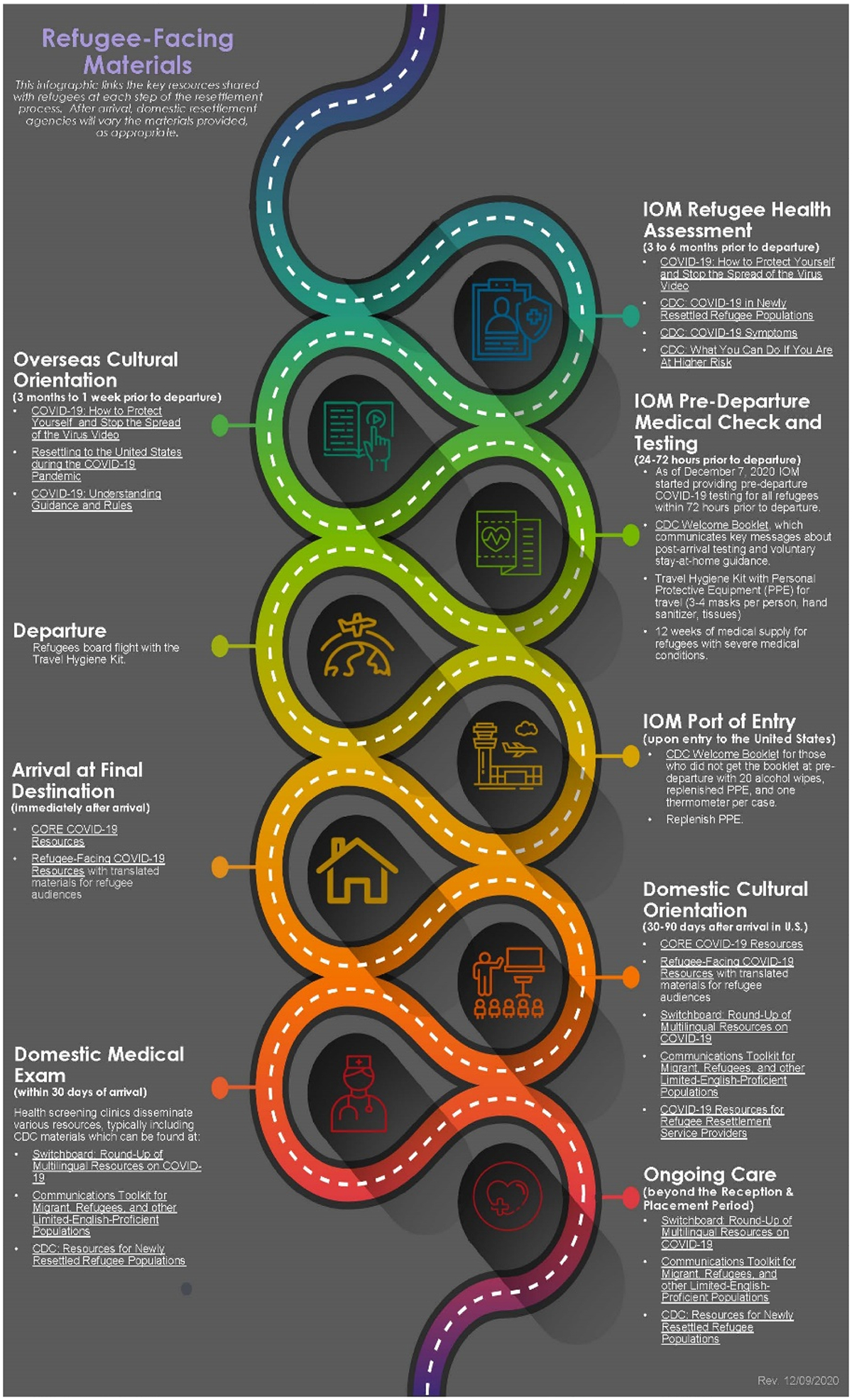
Refugee-facing materials developed by Bureau of populations, refugees, and Migration (Archived June 2022).

CORE and CDC produced several complementary communications resources and COVID-19 education documents for integration into domestic cultural orientation, a forum which provides recently arrived refugees with detailed lessons about what to expect upon arriving in the United States (Cultural Orientation Resource Exchange, 2021). With PRM guidance, CORE produced a toolkit of resources targeted to recently resettled refugees, detailing specific COVID-19 prevention measures in plain language and a low-literacy format. These multimodal resources, including fact sheets, frequently asked questions (FAQs), and podcasts, were translated into RIM specific – languages and reinforced key COVID-19 prevention messaging. Additionally, CDC provided public health guidance to partners, including clinicians and refugee health providers, as well as translated print resource materials, which could be used in the domestic cultural orientation. Finally, PRM led the development of the COVID-19 Refugee Health Resource Infographic (Figures 1 and 2), which offered a summary of key messages and resources provided to refugees at each step of the resettlement process. This infographic has been shared across multiple agencies’ websites and is updated as necessary.

Dissemination of COVID-19 communication resources was of critical importance to the work group, particularly in the early stages of the COVID-19 response, given the lack of targeted COVID-19 prevention resources for RIM communities. This dissemination process included sharing resources across the broad networks of the various federal agencies. These resources were also disseminated to state resettlement coordinators and local affiliates. Resource updates were amplified through weekly communications with the Association of Refugee Health Coordinators (ARHC), State Coordinators of Refugee Resettlement (SCORR), federal partner calls, and several webinars, conducted with the following groups: resettlement agency national and local affiliate staff, technical assistance partners, and domestic cultural orientation providers and leaders. Prior federal agency collaboration helped facilitate the smooth formation of this work group; already-established relationships served to enhance communication channels and improve resource sharing. We were not able to collect systematic data on the communication needs of refugees or the effectiveness of the created messages due to rapidly changing public health needs, especially early in the pandemic, and funding and staffing shortages in all partner groups. Thus, routine meetings in this collaboration facilitated sharing of the most up to date COVID-19 prevention health messaging and reinforced accountability on meeting deadlines, feedback loops, and further development of resources for refugees along the resettlement continuum. The meetings enabled partners to share key successes and challenges with refugee reception of targeted COVID-19 messaging at various touchpoints, so that local input could be integrated into the resource development process.

## Discussion

There were lessons learned through the evolution and progression of this work group. Increased communication and coordination between federal partners and international organizations enhanced the ability for agencies to effectively respond to communication needs from service providers and public health practitioners supporting refugees with changing pandemic conditions. Communication networks between various partners and their local networks were strengthened and service providers and public health practitioners were able to provide more concrete and direct feedback about their challenges. The work group capitalized on its diversity of addressing various elements of the health of refugees across the continuum of care by determining which agencies had optimal resources to complete various tasks most efficiently, given resource availability and competing work priorities. For example, to ensure timely information was disseminated for refugees in their preferred languages, CDC translated and published CDC-branded print resources, while CORE provided translation support for non-CDC branded materials. CDC formulated key public health messages for the Welcome Booklet, based on COVID-19 health messaging gaps that IOM identified for refugees in transit. IOM determined that refugees experienced increased vulnerabilities before, during, and after travel, so CDC designed and translated into 19 languages a COVID-19 prevention resource that addressed all three stages of resettlement and translated into 19 languages.

These resources established consistent COVID-19 prevention health messaging for refugees and resettlement partners, in a short and concise format, taking into consideration plain language, limited English proficiency levels to promote refugees’ comprehension of health information. Content for updates to the resources concerned both changes to public health guidance but also feedback from service providers in direct communication with refugee communities.

## Conclusion

This work group continued to meet quarterly from Spring 2020 until Fall 2022 and served as a coordination group for evolving COVID-19 health messaging and other emergency response efforts. As the pandemic evolved and new challenges emerged, the resources were updated to reflect the changing guidance. In addition to resource development, the group continued to cross-share and promote translated materials. The work group explored ways to utilize resources created (like the Welcome Booklet for Refugees) and consider opportunities to integrate this resource into an overarching health curriculum for U.S.-bound refugees through the U.S. Refugee Admissions Program. The work group federal agency and international partners will continue to stay engaged and work to meet the evolving needs of refugee communities across the continuum of care in the wake of the COVID-19 pandemic and beyond.

## Figures and Tables

**Table 1.** Interagency response to COVID-19 health messaging during early phase of pandemic: April–September 2020.

Agency	Gaps identified	Resource developed	Dissemination strategy
Bureau for population, refugees, and Migration (PRM)	How refugees can prevent the spread of COVID-19 at home and resources to reach out to resettlement agencies or clinicians, if needed.	COVID-19-Refugee-Health-resource-Infographic (Archived June 2022)	Webinars and Outreach to Local Resettlement Agencies (LRAs)
Cultural Orientation Resource Exchange (CORE)	Need to streamline health communication to resettlement partners; Integration COVID-19 into domestic cultural orientation curriculum	Resettling to the United States During the COVID-19 Pandemic Lesson Plan COVID-19: Understanding Guidance and Rules - Settle In (settleinus.org) Archived: COVID-19 Understanding, Guidance, and Rules Factsheet, Video, and Podcast Episode	Online refugee resource repository called Switchboard and outreach to LRAs
Centers for Disease Control and Prevention (CDC)	How to respond to partner COVID-19 public health needs quickly and effectively How to develop guidance related to newly arrived refugees	COVID-19 in Newly Resettled Populations Guidance (integrated into CDC’s Immigrant, Refugee, and Migrant Health Page COVID-19 Welcome Booklet (archived June 2022) but updated here: COVID-19 Information for Newcomers What You Can Do if You are at Increased Risk of Severe Illness from COVID-19 (archived) but factsheet integrated here: COVID-19 Information for Newcomers	Weekly partner updates to federal partners who disseminate to state and local health partners
International Organization for Migration (IOM)	Graphic materials regarding COVID-19 prevention measures before, during and after travel for refugees at higher risk of severe illness	Distribute COVID-19 Information for Newcomers to refugees before their departure to the USA	Resource provided to refugees at the overseas medical exams and the pre-departure medical checks
Office of Refugee Resettlement (ORR)	How to continue and adapt resettlement services impacted by COVID-19 related delays, such as domestic medical screening and telehealth appointments Need for COVID-19 translated health materials	FY 2020 Refugee Support Services COVID-19 Supplemental Allocation Dear Colleague Letter 21–05: COVID-19 Vaccinations and the Role of ORR Grantees DCL 21–08 COVID-19 Vaccination and Medical Screening	Resources shared through state refugee coordinators and state refugee health coordinators and networks
